# Effectiveness of active school transport interventions: a systematic review and update

**DOI:** 10.1186/s12889-017-5005-1

**Published:** 2018-02-01

**Authors:** Richard Larouche, George Mammen, David A. Rowe, Guy Faulkner

**Affiliations:** 1 Healthy Active Living and Obesity Research Group, Children’s Hospital of Eastern Ontario Research Institute, Ottawa, ON K1H 8L1 Canada; 2 Faculty of Health Sciences University of Lethbridge, 4401 University Drive, office M3049 Lethbridge, Alberta, T1K 3M4 Canada; 3 Centre for Addiction and Mental Health, Institute for Mental Health Policy Research, 1001 Queen St West, Toronto, ON M6J 1H4 Canada; 4 School of Psychological Sciences and Health, University of Strathclyde, 16 Richmond St, Glasgow G1 1XQ, Glasgow, UK; 5 School of Kinesiology, University of British Columbia, D H Copp Building 4606, 2146 Health Sciences Mall, Vancouver, BC V6T 1Z3 Canada; 6 Center for Hip Health and Mobility, Robert H.N. Ho Research Centre, 5th Floor, 2635 Laurel St, Vancouver, BC V5Z 1M9 Canada

**Keywords:** Active travel, Physical activity, Children, Safe routes to school, School travel plans, Walking school buses

## Abstract

**Background:**

Active school transport (AST) is a promising strategy to increase children’s physical activity. A systematic review published in 2011 found large heterogeneity in the effectiveness of interventions in increasing AST and highlighted several limitations of previous research. We provide a comprehensive update of that review.

**Methods:**

Replicating the search of the previous review, we screened the PubMed, Web of Science, Cochrane, Sport Discus and National Transportation Library databases for articles published between February 1, 2010 and October 15, 2016. To be eligible, studies had to focus on school-aged children and adolescents, include an intervention related to school travel, and report a measure of travel behaviors. We assessed quality of individual studies with the Effective Public Health Practice Project quality assessment tool, and overall quality of evidence with the Grades of Recommendation, Assessment, Development, and Evaluation (GRADE) approach. We calculated Cohen’s *d* as a measure of effect size.

**Results:**

Out of 6318 potentially relevant articles, 27 articles reporting 30 interventions met our inclusion criteria. Thirteen interventions resulted in an increase in AST, 8 found no changes, 4 reported inconsistent results, and 5 did not report inferential statistics. Cohen’s *d* ranged from −0.61 to 0.75, with most studies reporting “trivial-to-small” positive effect sizes. Three studies reported greater increases in AST over longer follow-up periods and two Safe Routes to School studies noted that multi-level interventions were more effective. Study quality was rated as weak for 27/30 interventions (due notably to lack of blinding of outcome assessors, unknown psychometric properties of measurement tools, and limited control for confounders), and overall quality of evidence was rated as low. Evaluations of implementation suggested that interventions were limited by insufficient follow-up duration, incomplete implementation of planned interventions, and limited access to resources for low-income communities.

**Conclusions:**

Interventions may increase AST among children; however, there was substantial heterogeneity across studies and quality of evidence remains low. Future studies should include longer follow-ups, use standardized outcome measures (to allow for meta-analyses), and examine potential moderators and mediators of travel behavior change to help refine current interventions.

**Trial registration:**

Registered in PROSPERO: CRD42016033252

**Electronic supplementary material:**

The online version of this article (10.1186/s12889-017-5005-1) contains supplementary material, which is available to authorized users.

## Background

Consistent evidence shows that children and adolescents who engage in active school transport (AST) are more physically active than those who travel by motorized vehicles [1, 2]. Cycling to and from school can also increase cardiovascular fitness [1] and is associated with a better cardiometabolic health profile [3]. At the population level, replacing motorized travel by AST could reduce exhaust and greenhouse gas emissions [4, 5]. Additional benefits of AST include positive emotions during the school trip [6], better way-finding skills [7] and superior school grades [8].

Despite these benefits, the prevalence of AST has decreased markedly during the last few decades in many countries [9–13]. To address this issue, many interventions have been implemented. Perhaps the most well-known is the Safe Routes to School (SRTS) program which has received over one billion dollars in funding from the US government [14]. Recent analyses concluded that New York City’s SRTS program led to a 33-44% reduction in injuries among school-aged children and the program was cost-effective even when disregarding any potential benefits related to increased physical activity and decreased congestion and pollution [15, 16]. In other jurisdictions, school travel plans (STP) have been implemented to address key barriers to AST at the local level, but often with limited funding [17–21]. Moreover, walking school buses (WSB) wherein children walk together on a set route with adult supervision have been implemented in many jurisdictions to address parental safety concerns [22, 23].

To our knowledge, Chillón and colleagues [24] published the first systematic review of the effectiveness of AST intervention among children and adolescents in 2011. While the included interventions were quite heterogeneous, most observed small increases in AST. However, quality of evidence for all interventions were rated as “weak” based on the Effective Public Health Practice Project (EPHPP) quality assessment tool for quantitative studies [25]. Moreover, none of the interventions examined the moderators and mediators of travel behavior change. A better understanding of moderators and mediators would enable researchers to understand what works for whom and why. We provide a comprehensive update on the effectiveness of AST interventions in children and youth that have been published over the last 6 years. We also aimed to review the literature on the moderators and mediators of AST interventions.

## Methods

### Search strategy

As our goal was to update the previous review [24], we replicated their search strategy. Databases searched included PubMed, Web of Science (SCI and SSCI), SPORTDiscus, the Cochrane library, and the National Transportation Library. The search terms addressed four main categories: *school-age children* (adolescen* OR child OR children OR youth OR student* OR pupil OR pupils) AND *active transportation* (bike OR bikers OR biking OR bicycl* OR cycle OR cycling OR cyclist* OR commute* OR commuting OR transportation OR travel*) AND *intervention* (intervention* OR implement* OR evaluat* OR change OR pilot OR project OR environment* OR engineer* OR encourage* OR planning OR impact OR “walk to school” OR “safe routes to school” OR “walking schoolbus” OR “walking school bus” OR “walking school buses”) AND *school*. Articles published between February 1, 2010 (the cut-off date of the previous review) and October 15, 2016 were considered eligible. Our review is registered in PROSPERO (CRD42016033252; see http://www.crd.york.ac.uk/PROSPERO/display_record.asp?ID=CRD42016033252).

### Inclusion and exclusion criteria

To be included in the review, studies had to: 1) have been conducted among children and adolescents (6-18 year olds); 2) focus on AST; 3) include an intervention; and 4) examine the effect of the intervention on a measure of active transportation or physical activity. Studies that did not meet all of these criteria were excluded. Language was not an exclusion criterion. Titles and abstract of all potentially relevant articles were screened by GM and GF. Full text copies of all articles that were not excluded at this stage of the review were then screened by GM and GF. Any discrepancy was resolved by consensus.

### Data extraction

The following data were extracted from each included study: lead author, country, a brief description of the intervention and its methodology, the effects on AST, the moderators and mediators examined, the effects on other outcomes and the types of strategies that were used based on the Safe Routes to School 6E model [14]. The 6 E’s are: 1) education (teaching students and community members about the different transportation options and ensuring they have the skills and know-how to be safe in traffic); 2) encouragement (using events, activities and incentives to promote AST); 3) engineering (making improvements to the built environment to increase safety); 4) enforcement (partnering with law enforcement to address traffic and crime concerns in the neighborhoods around schools and along school routes); 5) evaluation (assessing the effectiveness of the interventions); and 6) equity (ensuring that initiatives are benefiting all demographic groups). By definition, all studies that met our inclusion criteria have used evaluations, so this strategy was not extracted. Data extraction was done by RL and GM for a subsample of studies, and only by RL for the remainder. When relevant information was missing from included papers, we attempted to contact the lead author and/or the senior author.

### Quality assessment

To assess the methodological quality of each study, we used an adapted version of the EPHPP. This tool includes 6 components: 1) selection bias; 2) study design; 3) control for confounders; 4) blinding of participants and study staff; 5) validity and reliability of the data collection tools; and 6) withdrawals and drop-outs. Each component was rated as “weak”, “moderate” or “strong” based on standardized criteria, and then the number of weak ratings was tallied. Following the EPHPP approach, studies with zero weak ratings were rated as strong, studies with one weak rating were rated as moderate, and studies with at least two weak ratings were rated as weak. We retained the modifications proposed by Chillón and colleagues [24] to make the tool more suitable to studies in which the school is the unit of allocation. We also added a number of precisions to clarify the interpretation of the items. Our adapted EPHPP tool is available in Additional file 1. Quality assessment was first performed by RL and DR for a subsample of five studies. After consensus was attained for these studies, the remaining articles were assessed either by RL or DR. In case of doubt, the reviewer was asked to indicate the issue in an Excel spreadsheet and all issues were resolved by consensus among the two reviewers. Because blinding of participants was considered unfeasible in the context of most AST interventions, we present results both with and without the blinding component of the EPHPP. In addition, we assessed overall quality of evidence using the “Grades of Recommendation, Assessment, Development, and Evaluation” (GRADE) approach [26, 27]. Following this approach, randomized controlled trials begin as high quality evidence, but they may be downgraded based on limitations in the design and implementation, indirectness of evidence, unexplained heterogeneity of results, imprecision of estimates, and high probability of publication bias. Observational studies begin as low quality evidence, but may be upgraded if there are large effect sizes, a dose-response gradient, or if all plausible confounding would reduce the treatment effect [26, 27]. The overall quality of evidence was rated by consensus among the authors.

### Statistical analyses

Following the procedures of Chillón and colleagues [24], we computed Cohen’s *d* as a measure of effect size for each intervention. For interventions that included a control group, effect size was computed as the standardized mean difference of the changes in AST between the experimental and control groups. For those that included only an experimental group, it was computed between baseline and follow-up data. Additional file 2 provides comprehensive details on how effect sizes were computed for each intervention. Authors were contacted to obtain information required to calculate *d*. Following Cohen’s [28] guidelines, effect size was categorized as trivial (*d* < 0.2), small (*d* = 0.2), medium (*d* = 0.5), or large (*d* = 0.8). Due to the large methodological heterogeneity of the included studies (see Table 1), meta-analysis was considered inappropriate.

**Table 1:** Characteristics of the included interventions (*n* = 30)

Author, Country	Intervention and strategies	Methods	Effect on AST^a^	Effects on other outcomes	Moderators and mediators
Buckley et al. 2013 [29] [fall event] Idaho, USA	*Designated AST day*, aimed to encourage students and their families to practice AST on a specific day. Strategies: encouragement only.	Design: Observational case study | pre-post (during, 1 day after) Duration: 1 day. Sample: 2 primary schools. AST measure: observation counts (trips to school only, walking and cycling combined).	Relative increase in AST (101%) on the day; remained high one day following.	None examined.	None examined.
Buckley et al. 2013 [29] [spring event] Idaho, USA	*Designated AST day*, aimed to encourage students and their families to practice AST on a specific day. Strategies: encouragement only.	Design: Quasi-experimental | pre-post (during, 1 day after, 2 weeks after) Duration: 1 day. Sample: 3 primary schools (2 experimental, 1 control). AST measure: observation counts (trips to school only, walking and cycling combined).	Increase in AST sustained at 2-week follow-up relative to the control school (χ^2^ = 11.6; *p* = 0.009).	Parent escort increased by 333% on AST day (*p* < 0.001). Parent interviews suggested that the school journey is an opportunistic time to spend with their child.	None examined.
Buliung et al. 2011 [30] 4 Canadian provinces: Ontario, Alberta, British Columbia, Nova Scotia	*School Travel Planning,* a school-specific intervention aimed to increase AST through a collaborative stakeholder approach. Strategies engineering, education, enforcement and encouragement (varied between schools).	Design: Observational | pre-post (1 year). Sample: 12 primary schools, 1489 parent questionnaires. Duration: 1-year. AST measure: student classroom survey + parent questionnaire (trips to/from school, all active modes combined).	Student reported data indicated a modest increase in AST at follow-up (from 43.8 to 45.9%). *d* = 0.05. *P* value is unavailable (G. Faulkner, personal communication).	13% of parents reported driving less as a result of the intervention. According to parents the 3 most effective strategies were education, special events, and infrastructure improvements.	None examined.
Bungum et al. 2014 [31] Las Vegas, USA	*Designated AST day*, aimed to encourage students and their families to practice AST on a specific day. Strategies: encouragement only.	Design: Quasi-experiment | pre-2 post (during, 1-week) assessments Duration: 1-day event. Sample: 2 schools (1 experimental and 1 control) | 1336 students ages 5-11. AST measures: observation counts (trips to school only, all active modes combined).	Increase in the mode share of AST by 7.4 percentage points on the day of the event. AST was then significantly higher than in the control school (χ^2^ = 27.2; *p* < .001; *d* = 0.29). AST dropped to baseline rates at 1-week assessment.	No effect on the number of motor vehicles observed around the schools.	The increase in AST was larger in girls (χ^2^ = 13.5; *p* < .05) than boys (χ^2^ = 1.79; *p* = .056), but formal moderation analysis was not reported.
Christiansen et al. 2014 [32] Denmark	A comprehensive school-based intervention to improve non-curricular PA through changes of the physical and school environment supported by educational activities. Intervention schools were asked to have a policy targeting AST, to offer cycling safety education. Strategies: engineering, education, enforcement and policy.	Design: RCT | pre-post (2-year). Sample: 14 schools (7 experimental and 7 control) | 1014 students | ages 11-13. Duration: 2 years. AST measure: travel diary (trips to/from school, all active modes combined).	The prevalence of AST increased from 87.8% to 88.8% in the experimental group and from 84.3% to 85.3% in the control group with no difference between groups (*p* = 0.30; *d* = 0.13).	The intervention had no effect on perceived safety of the school route, parental encouragement of cycling and attitudes toward cycling. Note: improved cycling infrastructure was not implemented as planned due to limited funding.	Students reporting an unsafe route to school at baseline were more likely to use AST at follow-up in the intervention group compared to students with an unsafe route in the control group (OR = 2.69; 95% CI = 1.20–6.07). No interactions for gender, parent encouragement, distance, walkability and baseline AST.
Coombes et al. 2016 United Kingdom	A technology-based intervention (Beat the Street) aimed to increase AST via incentive-motivated approaches. Strategies: encouragement only.	Design: quasi-experimental | pre-2 post (7-weeks, 20-weeks) assessments. Duration: 9-week intervention. Sample: 2 schools (1 intervention and 1 control) | 80 students | ages 8-10. AST measure: travel diary (trips to/from school, all active modes combined).	At 7-week follow-up: no difference in AST between groups. At 20-week follow-up: 10% increase in AST in the intervention group and 7% decrease in control group (*p* = 0.056).	No difference in accelerometer counts per minute, but there was a smaller decline in MVPA in the experimental group (−15.1 vs. -23.3 min/day; *p* = 0.020).	Children who touched a Beat the Street box more often were more active (+3.5 min/day of MVPA for children who engaged in the intervention on the mean number of days, that is 14.5 days).
Crawford & Garrard, 2013 [34] Victoria, Australia [pilot schools]	The Ride2School Program, which consisted mostly of promotional activities with some infrastructure changes. Strategies: education, encouragement, and engineering.	Design: Quasi-experimental mixed methods study | pre-post (~1 year). Sample: 4 primary schools (2 control and 2 intervention) | participants were ages 10-13, but younger children were also counted in the observations. Duration: 9-12 months. AST measure: observation counts + student hands-up survey (trips to school only, all active modes combined).	Observation results show that AST increased significantly in the inner suburban pilot school and the outer suburban control school. Hands-up surveys show that AST increased in the inner suburban pilot school and no changes were found in the other schools.	Qualitative data suggest that the program was easier to implement within a school that was smaller, more established, with a culture that was accepting and enthusiastic about AST, in an area of higher density and lower car use, with more infrastructure improvements and a more “hands-on” approach from the Coordinator.	Results differed by level of urbanization (see “effects on AST” column).
Crawford & Garrard, 2013 [34] Victoria, Australia [program schools]	The Ride2School Program, which consisted mostly of promotional activities, without infrastructure changes. Strategies: education and encouragement.	Design: Observational mixed methods study | pre-post (6 months). Sample: 13 primary schools. Duration: 6 months. AST measure: student hands-up survey and parent survey (trips to school only).	Parent surveys: increase in cycling (from 13.9 to 15.9%) and decrease in scooter or skateboard use (from 6.2 to 5.0%). The proportion of parents reporting ≥1 active trips increased (adjusted OR = 1.67; 95% CI = 1.04-2.68). Child surveys: decrease in scooter or skateboard use (from 7.2 to 4.9%) and a decrease in AST (from 51.1 to 48.7%). The latter was no longer significant after adjustment. *d* = 0.04 for parent report and −0.06 for child report.	Qualitative data suggest that program implementation varied between schools and that the program was more effective when school communities were highly motivated, when secure bike storage facilities were offered, when all active modes were promoted equally by dynamic school staff.	None examined.
Ducheyne et al. 2014 [35] Belgium	*Cycling training*, aimed to increase cycling skills and encourage uptake of cycling. Strategies: education only.	Design: RCT | pre-post (1-week, 5-month) post assessments. Sample: 3 primary schools (2 distinct experimental groups and 1 control group) | 94 students | age 9.3 ± 0.5. Duration: 4 sessions (45 min each). AST measure: parent reported (to/from school, time spent cycling only).	Changes in weekly time spent cycling did not differ between intervention and control group (F = 1.9; *p* > 0.05) Effect sizes: intervention vs. control group: *d* = 0.46; intervention + parent vs. control: *d* = 0.03.	Children’s cycling skill score increased significantly more in the intervention group from pre to post (F = 16.9; *p* < 0.001) and from pre to 5-months follow-up (F = 16.8; *p* < 0.001) compared to the control group. No intervention effects for parental attitudes.	None examined.
Goodman et al. 2016 [36] United Kingdom	*Bikeability*, a national cycle training program for children and adults. Strategies: education only.	Design: retrospective natural experiment. Sample: 3336 children whose school either had offered (*n* = 2563) or had not yet offered Bikeability (*n* = 773) | age 10-11. Duration N/A. AST measure: parent-reported frequency of cycling (at least once a week vs. less), and whether cycling was children’s usual travel mode to school.	Children attending schools that had offered Bikeability were not more likely to cycle at least once a week (OR = 0.99; 95% CI = 0.89-1.10) and to cycle to school (OR = 0.73; 95% CI = 0.41-1.29). Children who received Bikeability training were more likely to cycle at least once a week (OR = 1.26; 95% CI = 1.16-1.37).	Children attending schools that had offered Bikeability were much more likely to have completed the program (68% vs. 28%; *p* < 0.001).	Children’s participation in Bikeability was identified as a potential mediator of the relationship between school exposure to Bikeability and cycling frequency; however, no main effect was observed.
Gutierrez et al. 2014 [37] Miami, USA	Implementation of crossing guards & AST awareness campaign. Strategies: education, equity.	Design: Quasi-experimental | pre-post. Sample: 58 intersections near elementary schools (24 where a new crossing guard was present and 34 control). AST measure: observation counts (to school only, walking and cycling examine separately and combined).	The number of pedestrian and cyclists did not change following the addition of crossing guards (*p* > 0.05; *d* = 0.03).	Safety: increase in students’ use of supervised routes with a moderate effect size (partial η^2^ = 0.008). No changes in parental attitudes regarding AST safety.	None examined.
Henderson et al. 2013 Atlanta, USA	*Safe Routes to School*, a comprehensive, federally-funded program in the US designed to increase AST through non- infrastructure and infrastructure strategies. Strategies: education, encouragement, and engineering.	Design: Observational | pre-post. Sample: one primary school (658 students). Duration: 2 years. AST measure: parent-reported (to/from school); all active modes combined.	The prevalence of AST increased from 18% to 42% in the morning trip (*p* < 0.0001; d = 0.66), and from 18% to 23% in the afternoon (NS; d = 0.17).	Parental perception about the health benefits, perception that the school strongly encouraged AST, and enjoyment of walking/biking to school increased significantly (all *p* < 0.01).	None examined.
Hinckson et al. 2011a New Zealand	*School Travel Planning*, a school-specific intervention aimed to increase AST through a collaborative stakeholder and multi-strategic approach. Strategies: engineering, education, enforcement, encouragement, and policy interventions.	Design: Observational | pre-post (1 to 2 years). Sample: 33 primary schools | 13,631 students. Duration: 1-2 years. AST measure: student reported (to school only, all active modes combined).	AST increased by 5.9 ± 6.8%. *d* = 0.45.	None examined.	Larger increase in AST with longer follow-up period. Longer follow-up period, smaller school roll and higher pre-intervention rate of AST predicted higher rates of AST at follow-up.
Hinckson et al. 2011b [18] New Zealand	*School Travel Planning*, a school-specific intervention aimed to increase AST through a collaborative stakeholder and multi-strategic approach. Strategies: engineering, education, enforcement, encouragement, and policy interventions.	Design: Observational | pre-post (1-, 2- and 3-year). Sample: 56 primary schools | 57,096 students | grades K-5. Duration: 3 years. AST measure: student reported (to school only, all active modes combined).	There was an increase in AST by the 3rd year of implementation (from 40.5 to 42.2%; OR = 2.65; 95% CI = 1.75-4.02). *d* after 1, 2, and 3 years = 0.17, 0.51 and 0.54 respectively.	None examined.	Larger increase in AST with longer follow-up period. The program was more effective in older students, in smaller schools and in the city of Auckland, but it was less effective in low SES schools (all *p* < 0.05).
Hoelscher et al. 2016 [39] Texas, USA	*Safe Routes to School*, a comprehensive, federally-funded program in the US designed to increase AST through infrastructure and non- infrastructure strategies. Strategies: education and encouragement; some schools received infrastructure improvements (engineering).	Design: Quasi-experimental | pre-post (3 year). Sample: grade 4 students from 78 elementary schools. Schools were allocated to either of 3 conditions: “infrastructure” (*n* = 23), “non-infrastructure” (*n* = 21) or control group (*n* = 34). Duration: 3 years. AST measure: student reported (to/from school, all active modes combined).	Infrastructure and non-infrastructure schools had significantly higher rates of AST in the morning (*p* = 0.024 and 0.013 respectively) and non-infrastructure schools had significantly higher overall AST relative to control schools (*p* = 0.036). However, differences between groups attenuated over time.	Students from both infrastructure and non-infrastructure schools had higher AST-related self-efficacy, and a similar finding was noted in infrastructure schools for parents. Students in non-infrastructure schools reported engaging in PA on more days than students from comparison schools. Parents from all types of schools perceived worse walkability and bikeability in their neighborhoods and schools over time.	None examined.
Hunter et al. 2015 [40] London, England Reading, England Vancouver, Canada	International school competition, aimed to increase AST via incentive-motivated approaches. Strategies: encouragement only.	Design: Observational Mixed-Methods (4-week). Sample: 12 primary and secondary schools | 3817 Students | 9-13 year olds. Duration: 4 weeks. AST measure: Objective swipe card technology and child reports (to/from school, walking only).	The percentage of walking trips measured by the swipe card decreased over the 4-week measurement period from 29 to 12%. However, at baseline 77% of children stated that they walked to school at least once in the past week and this proportion was 86% at follow-up. *d* = −0.61 with swipecard methodology and 0.34 with self-report.	Children’s attitudes: perceived the intervention to help physical and mental health. Adult attitudes: 91% of parents and 72% of teachers surveyed stated that they thought the competition had encouraged children to spend more time walking with their friends. This was corroborated with focus groups data.	None examined.
Johnson et al. 2016 [41] England [Bikeability school travel survey]	*Bikeability*, a national cycle training program for children and adults. Strategies: education only.	Design: retrospective case-control analysis. Sample: 1345 year 5 and 6 students. Duration: N/A. AST measure: frequency of cycling to school as well as cycling in general (child report).	Students who received Bikeability were more likely to cycle to school (OR = 2.25; 95% CI = 1.83-3.52). *d* = 0.45.	Students who received Bikeability did not report more cycling in general (OR = 1.01; 95% CI = 0.75-1.38). Year 6 students who received Bikeability expressed greater confidence (OR = 1.81; 95% CI = 1.26-2.59).	None examined
Johnson et al. 2016 [41] England [CensusAtSchool]	*Bikeability*, a national cycle training program for children and adults. Strategies: education only.	Design: retrospective case-control analysis. 1745 year 7-9 students. Duration: N/A. AST measure: frequency of cycling to school as well as cycling in general (child report).	Students who received Bikeability were more likely to cycle to school (OR = 1.60; 95% CI = 1.17-2.21). *d* = 0.26.	Students who received Bikeability were more likely to report cycling ≥30 min in the past week (OR = 1.27; 95% CI = 1.07-1.51).	None examined.
Mammen et al. 2014a [19] Canada	*School Travel Planning,* a school-specific intervention aimed to increase AST through a collaborative stakeholder and multi-strategic approach. Strategies (varied across schools): education, encouragement, engineering, enforcement.	Design: retrospective analysis (1-year following implementation). Sample: 53 primary schools, 7827 questionnaires. Duration: 1 year. AST measure: parent questionnaire (to/from school, all active modes combined).	17% of the parents reported driving less as a result of the intervention. Of these, about 83% reported switching from driving to AST. No baseline data available and no hypothesis test performed.	None examined.	Parents of older students, those living <3 km away from school, attending urban and suburban schools, and attending medium-SES schools were more likely to report less driving.
Mammen et al. 2014b [64] Canada	*School Travel Planning,* a school-specific intervention aimed to increase AST through a collaborative stakeholder and multi-strategic approach. Strategies (varied across schools): education, encouragement, engineering, enforcement.	Design: Observational | pre-post (1-year). Sample: 53 primary schools. Duration: 1 year. AST measure: Student reported hands-up survey (to/from school, all active modes combined).	Baseline and follow-up data showed that 27% and 31% of children engaged in AST to and from school, with no intervention effect. *d* = −0.02 for morning trip and 0.01 for afternoon trip. Changes in AST ranged from a 26% decrease and a 23% increase across schools.	None examined.	Schools that collected baseline data in the Fall (i.e., September) and follow-up data in Winter (i.e., February) observed a 5% decrease in AST (B = −5.36, *p* < .05).
McDonald et al. 2013 [42] Oregon, USA	*Safe Routes to School*, a comprehensive, federally-funded program in the US designed to increase AST through non- infrastructure and infrastructure strategies. Strategies: engineering, education, encouragement, and enforcement (varied between schools).	Design: Quasi-experimental | pre-post. Sample: 9 primary schools and 5 middle schools (includes 9 experimental and 5 control schools). Duration: up to 4 years. AST measure: student reported (to/from school, walking and biking combined).	Education + encouragement were associated with increases in walking and biking by 2 and 5 percentage points respectively. Augmenting education programs with engineering improvements was associated with increases in walking and biking of 5-20 percentage points.	None examined.	More comprehensive programs were associated with greater increases in AST (see “effect on AST” column).
McDonald et al. 2014 [43] Florida, Oregon, Texas, District of Columbia, USA	*Safe Routes to School*, a comprehensive, federally-funded program in the US designed to increase AST through non- infrastructure and infrastructure strategies. Strategies: engineering, education, encouragement, and enforcement (varied between schools).	Design: Quasi-experimental | pre-post (5-year). Sample: 801 schools of which 83% were elementary schools (includes 378 experimental and 423 control schools) | 65,289 students. Duration: 5 years. AST measure: student reported (to/from school, walking and biking combined).	Relative to control schools, each year of participation in SRTS was associated with a 1.1% increase in AST (*p* = 0.002; *d* = 0.019). Engineering improvements led to a 3.3 percentage point increase in walking and biking (*p* = 0.031; *d* = 0.12), while education + encouragement interventions led to a 0.9 percentage point increase per year (*p* = 0.025; *d* = 0.15).	None examined.	More comprehensive programs were associated with greater increases in AST (see “effect on AST” column).
McMinn et al. 2012 [44] Glasgow, Scotland	*Travelling Green*, a 6-week school based intervention aimed to increase AST via teacher lesson plans and student packs (e.g., material to set walking goals, record behavior). Strategies: education and encouragement.	Design: Quasi-experimental | pre-post. Sample: 5 primary schools (2 experimental and 1 control) | 166 students | ages 8-9. Duration: 6 weeks. AST Measure: Step counts and MVPA measured by Actigraph accelerometers during the trips to and from school.	Intervention group had smaller decreases in mean steps (−47 vs. -205) and seconds of MVPA (−33 vs. -85) during the morning trip. Opposite results on the afternoon trip for steps (−222 vs. -120) and MVPA (−125 vs. -59). *d* < 0.1 for changes in steps and MVPA during the school trip.	Children who received the intervention showed a smaller decline in daily step counts (−901 vs. -2528; *d* = 0.52) and time spent engaging in MVPA (−429 vs. -1171 s; *d* = 0.46).	None examined.
Mendoza et al. 2011 [45] Texas, USA	*Walking School Bus*, aimed to increase AST by having children walking in adult-supervised groups. Strategies: encouragement only.	Design: RCT | data collected before and in 4th & 5th week of intervention. Sample: 8 primary schools (4 experimental and 4 control) | 149 students | average age = 10 years. Duration: 5 weeks. AST measure: Student reported (to/from school, all active modes combined).	In the intention-to-treat analyses, intervention children increased their weekly percent AST from 23.8% ± 9.2% at baseline to 54.0% ± 9.2% at follow-up, whereas control children decreased their weekly percent AST from 40.2% ± 8.9% to 32.6% ± 8.9% (*p* < .0001; *d* = 0.40).	Intervention children increased their MVPA from 46.6 ± 4.5 to 48.8 ± 4.5 min/day while controls decreased theirs from 46.1 ± 4.3 to 41.3 ± 4.3 min/day (*p* = .029; *d* = 0.18).	Acculturation (*p* = .014) and parent outcome expectations (*p* = .025) were both associated with increased AST. Parent self-efficacy was positively associated with AST (*r* = 0.182; *p* = .032).
Østergaard et al. 2015 [46] Denmark	*Safe and Secure Cycling to school,* aimed to increase cycling behaviors through a multicomponent cycling promotion program. Strategies: encouragement, enforcement, education and engineering.	Design: Quasi-experiment | pre-post (1-year) assessments. Sample: 25 primary schools (13 experimental and 12 control) | 2401 students | 4th & 5th grade students. Duration: 1 year. AST Measure: Student reported number of cycling trips to/from school in the past week (range = 0-10).	Change in the number of cycling trips to/from school were not significant (B = 0.15 trips; 95% CI = −0.25; 0.54). *d* = 0.02.	Cardiorespiratory fitness decreased in the intervention group relative to the control group (B = −1.45 ml O_2_·kg^−1^·min^−1^; *p* < 0.0001). No change in recreational cycling, overall PA, BMI and obesity.	None examined.
Sayers et al. 2012 [47] Columbia, USA	*Walking School Bus*, aimed to increase AST by having children walking in adult-supervised groups. Strategies: encouragement only.	Design: Case control analysis where the researchers compared accelerometry-measured PA between WSB participants and non-participants. Sample: 3 primary schools | 77 students | ages 8-9. Duration: 1 week.	None.	Percentage of time spent in MVPA did not differ between WSB participants and controls (all *p* ≥ 0.17). *d* = −0.32. The age-related gradient in MVPA was attenuated in WSB participants.	None examined.
Stewart et al. 2014 [48] Florida, Mississippi, Washington, Wisconsin, USA	*Safe Routes to School*, a comprehensive, federally-funded program in the US designed to increase AST through non- infrastructure and infrastructure strategies. Strategies: engineering, education, encouragement, and enforcement (varied between schools).	Design: Observational | pre-post. Sample: 53 primary schools with SRTS projects. Duration: 5 years. AST measure: student hands-up survey or observation counts (travel to school only). Assessed walking and biking separately and combined.	At the school level, AST increased from 12.8% to 19.8% (*p* < .001; *d* = 0.50); walking from 8.8% to 13.3% (*p* < .001; *d* = 0.46); cycling from 2.0% to 3.2% (*p* = 0.085; *d* = 0.32).	None examined.	Smaller changes in cycling in schools that had higher levels of cycling at baseline (*r* = −0.40; *p* = 0.009). Were not associated with changes in AST: funding, number of students/schools per project, project type, intervention strategies, school level, enrollment, % of students eligible for free/reduced cost meals, and characteristics of the school neighborhood.
Vanwolleghem et al. 2014 [49] West-Flanders, Belgium	*A drop-off spot* (500-800 m distance from school) was organized that included teacher supervision on the walk to/from the designated area. Strategies: encouragement only.	Design: Observational | data collected before and during intervention. Sample: 2 primary schools | 58 students | ages 6-12 (mean = 9.7 ± 1.6 years). Length: 1 week. AST measure: child reported the number of active trips using the drop-off spot in a diary.	The number of reported active trips per week increased from 1 to 3 (χ^2^ = 52.9; *p* < 0.001; *d* = 1.00).	Pedometer-determined step counts before/after school hours increased significantly (+732 step counts/day; χ^2^ = 12.2; *p* < 0.001), but not daily step counts (*p* = 0.16). Positive perception of the intervention by principals and parents, but teachers expressed doubts about future implementation.	None examined.
Villa-González et al. 2016 [50] Spain	*Intervention* aimed in increasing AST through changing children safety perceptions and attitudes. Strategies: education and encouragement.	Design: Quasi-experimental | pre-post (6 months). Sample: 5 primary schools (3 experimental and 2 controls) | 206 students | ages 8-11. Length: 6 months. AST measure: student-reported number of walking and cycling trips in the previous week (range = 0-10 trips).	Increase in the frequency of active trips in intervention schools (0.6 ± 0.2) relative to control schools (−0.4 ± 0.3) [*p* = 0.019; *d* = 0.40]. When examining travel modes separately, significant changes were only observed for walking.	None examined.	None examined.
Xu et al. 2015 [51] China	*Click Obesity Study,* a multicomponent lifestyle childhood obesity prevention program aimed to enhance lifestyle behaviors. Strategies (for lifestyle behavior change in general): education, encouragement.	Design: RCT | pre-post (1 year). Sample: 8 primary schools (4 intervention and 4 control) | 1182 students | 4th grade. Length: 1 year. AST measure: Student reported school travel mode (to/from school, walking and cycling combined).	Participants in intervention schools were more likely to change their travel mode to walking or cycling to school (OR = 2.24, 95% CI = 1.47-3.40; *d* = 0.45) relative to those in the control schools.	Intervention participants were more likely to show a ≥ 0.5 kg/m^2^ decrease in BMI (OR = 1.44, 95% CI = 1.10-1.87), to increase the frequency of jogging or running (OR = 1.55, 95% CI = 1.18-2.02), and to decrease TV/computer use (OR = 1.41, 95% CI = 1.09-1.84) and red meat consumption (OR = 1.50, 95% CI = 1.15-1.95).	None examined.

Characteristics are reported at the intervention level because some papers reported the findings of two interventions

*AST* active school transportation, *MVPA* moderate-to-vigorous physical activity, *NS* non-significant, *OR* odds ratio, *PA* physical activity, *SES* socio-economic status, *SRTS* Safe Routs to School, *STP* school travel planning, *WSB* walking school bus

^a^Details on the calculation of standardized effect sizes (Cohen’s *d*) are provided in Additional file 2

## Results

**Fig. 1:**
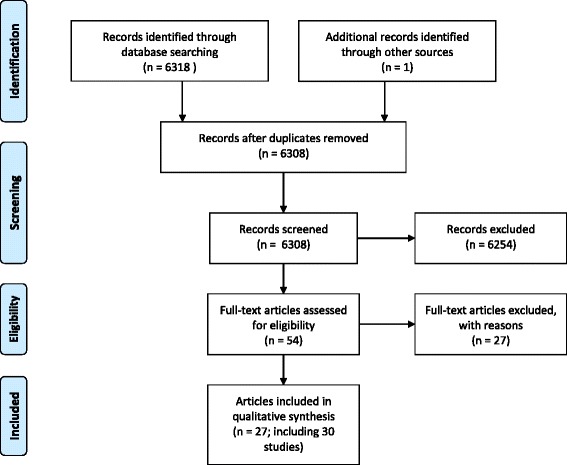
Flow of articles in the review process

The flow of papers in the review process is depicted in Fig. 1. Overall, 6318 papers were identified by the search including 2339 in PubMed, 1555 in Web of Science, 377 in Cochrane, 882 in SPORTDiscus, and 1165 in the National Transportation Library. One paper was identified from the authors’ personal libraries. All abstracts were screened, and 54 papers were found to be potentially eligible for inclusion. After a thorough selection process, 27 papers were excluded due to the following exclusion criteria: no/ineligible intervention, *n* = 17; no measure of physical activity or AST, *n* = 8; review article, *n* = 2. A total of 27 papers, reporting on 30 different interventions, were included for analyses [17–20, 29–51]. Results are presented at the intervention level because three papers reported the findings of two different interventions. Specifically, Buckley et al. [29] included a fall event without control group and a spring event with control group, Crawford and Garrard [34] included a pilot study with control schools (pilot schools) and a main study without control group (program schools), and Johnson et al. [41] reported case-control analyses using data from two different surveys conducted in distinct populations (Bikeability and CensusAtSchool). Eleven interventions were conducted in the US, five in the UK, three in Canada, two in Australia, Belgium, Denmark and New Zealand, and one in Spain and China. Another intervention was conducted simultaneously in Canada and the UK.

### Characteristics of interventions

Of these interventions, six evaluated Safe Routes to School (SRTS) interventions [38, 39, 42, 43, 46, 48], seven evaluated school travel plan (STP) projects [17–20, 30, 34], and two examined stand-alone walking school buses (WSB) schemes [45, 47]. Four interventions focused on the effects of bicycle training programs [35, 36, 41], five examined the effects of stand-alone events or contests [29, 31, 33, 40], and two were multi-component interventions that examined, among other things, changes in AST following the intervention [32, 51]. Others included two studies examining the effect of curriculum-based programs on AST [44, 50], one intervention using a drop-off spot from which driven children could walk to school with adult supervision [49], and an investigation of the effect of deploying crossing guards on travel behaviors [37]. Included studies assessed AST in a variety of ways including classroom hand-up surveys [17, 18, 20, 34, 42, 43, 48], child surveys and diaries [32, 33, 41, 45, 46, 49–51], parent surveys [19, 30, 34–36, 38], direct observation [29, 31, 32, 37, 48], using a swipe card technology [40] or by recording accelerometer steps taken during the school journey [44]. One study compared accelerometry-measured PA among participants in a WSB and non-participants [47]. Moreover, there was substantial heterogeneity in how AST was operationalized (e.g., travel mode on the day of the survey, usual travel mode, frequency of AST, etc.) and whether different active modes were assessed separately or pooled together (Table 1).

The majority of interventions focused on the elementary school setting. Only three studies included some secondary school students [40, 41, 43].The target sample size of included interventions ranged from 80 to 65,289 students. Schools were randomized to an intervention or a control group in four interventions [32, 35, 45, 51]. Of the remaining interventions, 11 used a pre-post design without a control group [17, 18, 20, 29, 30, 34, 38–40, 48, 49], 10 were quasi-experimental studies with a control group [29, 31, 33, 34, 37, 42–44, 46, 50], four were retrospective case-control studies [36, 41, 47], and one was a retrospective study [19]. A detailed description of the interventions and their main results is provided in Table 1.

### Quality assessment

Quality ratings are shown in Table 2. For individual components of the EPHPP, the proportion of weak ratings was 3.3% for study design, 30.0% for withdrawals and dropouts, 56.7% for selection bias, 60.0% for control for confounders, 66.7% for data collection methods, and 100% for blinding. Following Chillón and colleagues’ [24] modifications of the EPHPP, four studies were rated “non-applicable” for withdrawals and dropouts because participants were recruited after the intervention occurred and could not have dropped out. No study reported that outcome assessors or participants were blinded, and only two studies discussed blinding and specified that it was not feasible in their intervention [35, 45]. In analyses that included the blinding component of the EPHPP tool, only three studies were rated as “moderate” [32, 39, 45], and the remainder were rated as “weak”. In a sensitivity analysis that excluded the blinding component, study quality was rated as weak for 21 interventions [19, 20, 29–31, 33, 34, 36, 38, 40–43, 46, 48–51], moderate for six interventions [17, 18, 35, 37, 44, 47], and strong for three interventions [32, 39, 45]. While our review included some randomized controlled trials, most individual studies were rated as “weak” and very serious limitations in the design and implementation of interventions were noted, as mentioned above. Therefore, we attributed a low grade for the overall quality of evidence.

**Table 2:** Quality assessment of active school transportation interventions

Lead author (year)	Selection bias	Study design	Control for confounders	Blinding	Data collection	Withdrawals and dropouts	Global rating	Global rating without blinding
Buckley (2013) [fall event]	Weak	Moderate	Weak	Weak	Weak	Strong	Weak	Weak
Buckley (2013) [spring event]	Weak	Moderate	Strong	Weak	Weak	Strong	Weak	Weak
Buliung (2011)	Weak	Moderate	Weak	Weak	Weak	Weak	Weak	Weak
Bungum (2014)	Weak	Moderate	Weak	Weak	Weak	Strong	Weak	Weak
Christiansen (2014)	Strong	Strong	Strong	Weak	Moderate	Moderate	Moderate	Strong
Coombes (2016)	Weak	Moderate	Weak	Weak	Weak	Strong	Weak	Weak
Crawford (2013) [pilot]	Weak	Strong	Strong	Weak	Weak	Strong	Weak	Weak
Crawford (2013) [program]	Weak	Moderate	Weak	Weak	Weak	Strong	Weak	Weak
Ducheyne (2014)	Moderate	Strong	Strong	Weak	Weak	Strong	Weak	Moderate
Goodman (2016)	Moderate	Moderate	Strong	Weak	Weak	Weak	Weak	Weak
Gutierrez (2014)	Moderate	Strong	Strong	Weak	Weak	Strong	Weak	Moderate
Henderson (2013)	Moderate	Moderate	Weak	Weak	Weak	Weak	Weak	Weak
Hinckson (2011a)	Moderate	Moderate	Weak	Weak	Moderate	Moderate	Weak	Moderate
Hinckson (2011b)	Moderate	Moderate	Weak	Weak	Moderate	Strong	Weak	Moderate
Hoelscher (2016)	Moderate	Moderate	Strong	Weak	Strong	Strong	Moderate	Strong
Hunter (2015)	Weak	Moderate	Weak	Weak	Weak	Weak	Weak	Weak
Johnson (2016) [Bikeability]	Weak	Moderate	Weak	Weak	Weak	N/A	Weak	Weak
Johnson (2016) [CensusAtSchool]	Weak	Moderate	Weak	Weak	Weak	N/A	Weak	Weak
Mammen (2014a)	Weak	Weak	Weak	Weak	Weak	N/A	Weak	Weak
Mammen (2014b)	Moderate	Moderate	Weak	Weak	Strong	Weak	Weak	Weak
McDonald (2013)	Weak	Moderate	Weak	Weak	Moderate	Weak	Weak	Weak
McDonald (2014)	Weak	Moderate	Strong	Weak	Weak	Weak	Weak	Weak
McMinn (2012)	Moderate	Moderate	Weak	Weak	Strong	Strong	Weak	Moderate
Mendoza (2011)	Moderate	Strong	Strong	Weak	Strong	Strong	Moderate	Strong
Østergaard (2015)	Weak	Moderate	Weak	Weak	Weak	Moderate	Weak	Weak
Sayers (2012)	Weak	Moderate	Strong	Weak	Strong	N/A	Weak	Moderate
Stewart (2014)	Moderate	Moderate	Weak	Weak	Weak	Weak	Weak	Weak
Vanwolleghem (2014)	Weak	Moderate	Weak	Weak	Strong	Strong	Weak	Weak
Villa-Gonzalez (2016)	Moderate	Moderate	Strong	Weak	Weak	Weak	Weak	Weak
Xu (2015)	Weak	Strong	Strong	Weak	Weak	Strong	Weak	Weak

Quality assessment was conducted with a modified version of the Effective Public Health Practice Project quality assessment tool for quantitative studies (EPHPP, 2003), which is provided in Additional file 1. Following EPHPP guidelines, studies with no weak ratings are rated “strong”, studies with one weak rating are rated “moderate” and studies with more than one weak rating are rated “weak”. Considering that blinding of participants may not be feasible in the context of AST interventions, global ratings with and without the blinding component of the EPHPP are presented

### Intervention effectiveness

Overall, 13 interventions resulted in a statistically significant increase in AST [18, 29, 31, 38, 41, 42, 45, 47–51] while eight reported no changes in AST [20, 32, 33, 35, 37, 43, 46, 47]. Of the latter studies, McMinn et al. [43] reported a smaller seasonal decline in PA among children in their intervention group, and this can be viewed as a positive finding given that PA typically declines during the fall and winter. Five interventions did not include an hypothesis test for changes in AST [17, 19, 29, 30, 40]. The remaining studies reported inconsistent or conflicting results. Specifically, in their pilot study, Crawford & Garrard [34] reported a significant increase in AST in their inner suburban school, but no change in their outer suburban school relative to the control group. In their “program” phase, they reported a significant increase in AST in experimental schools based on parent surveys after adjusting for confounders, but their child surveys indicated no change in AST after statistical adjustment. Goodman and colleagues [36] reported that children attending a school that had offered the Bikeability program did not cycle more frequently; however, those who actually took part in Bikeability did cycle more frequently, suggesting that parents/children interested in cycling may have self-selected to participate. Finally, Hoelscher et al. [39] observed that while intervention schools had higher rates of AST over the 4-year study period, the differences between groups waned over time.

Details on the computation of effect sizes (Cohen’s *d*) are provided in Additional file 2. Cohen’s *d* varied markedly across interventions with a range of −0.61 to 0.75. Effect size could not be calculated for five interventions, including two that provided only follow-up data [19, 42], and three that provided insufficient data to allow for computation of *d* [29, 39]. Effect size was rated as trivial for 10 interventions [17, 20, 30, 32, 34, 36, 37, 43, 46, 47], small for eight interventions [31, 33, 39, 43, 48, 50, 51], and medium for one intervention [49]. Data from Hinckson et al. [18] indicate a trivial effect size after 1 year of follow-up, but a medium effect size after 2 or 3 years. Henderson and colleagues’ [38] SRTS intervention yielded a medium effect size for the morning trip and a trivial effect size for the afternoon trip. Data from Hunter et al. [39] indicated a medium decrease in AST as estimated with the swipe card methodology, but a small increase for self-reported AST. In Crawford and colleagues’ [34] pilot program, there was a small effect size for the inner suburban school and a trivial one for the outer suburban school. In the 3-group intervention by Ducheyne et al. [35], there was a small effect size when comparing the intervention and control groups, but a trivial effect size when comparing the intervention + parent (which targeted parents in addition to children) vs. the control group. Finally, data from McMinn et al. [44] suggest a small effect size for changes in minutes of moderate-to-vigorous PA per day, but a moderate effect size for changes in steps/day although both effect sizes were similar (*d* = 0.46 and 0.52 respectively); however, effect size was trivial for changes in steps and MVPA during the school trip. Table 3 summarizes effect sizes by type of intervention; however, no clear pattern is evident.

**Table 3:** Effect size of active school transportation interventions stratified by intervention type

	Measure of effect size	Cohen’s d
Safe Routes to school		
Henderson (2013)	Change in prevalence of AST (morning trip/afternoon trip)	0.66/0.17
McDonald (2014)	Change in prevalence of AST	0.19
Østergaard (2015)	Change in number of weekly AST trips	0.02
Stewart (2014)	Change in prevalence of AST	0.28
School travel planning		
Buliung (2011)	Change in prevalence of AST	0.05
Crawford (2013)	Change in prevalence of AST – inner suburban pilot school (direct observation/hands-up survey)	0.27/0.30
Crawford (2013)	Change in prevalence of AST – outer suburban pilot school (direct observation/hands-up survey)	−0.12/0.04
Crawford (2013)	Change in prevalence of AST in the program schools (parent report/child report)	0.04/-0.06
Hinckson (2011a)	Change in prevalence of AST	0.14
Hinckson (2011b)	Change in prevalence of AST according to length of follow-up (1 year/2 years/3 years)	−0.17; 0.51; 0.54
Mammen (2014b)	Change in prevalence of AST (morning trip/afternoon trip)	−0.02; 0.01
Walking school buses		
Mendoza (2011)	Change in percentage of trips using AST	0.40
Sayers (2012)	Difference in % of time spent in MVPA	−0.32
Cycle training		
Ducheyne (2014)	Change in weekly time spent engaging in AST (intervention vs. control group/intervention + parent vs. control group)	0.46/0.03
Johnson (2016)	Difference in odds of cycling to school between trained and untrained children (Bikeability survey)	0.45
Johnson (2016)	Difference in odds of cycling to school between trained and untrained (CensusAtSchool survey)	0.26
Goodman (2016)	Difference in odds of cycling to school between trained and untrained (school level/individual level)	−0.17; 0.18
Special events		
Bungum (2014)	Change in number of students engaging in AST	0.29
Coombes (2016)	Change in proportion of trips using AST at 7-week and 20-week follow ups respectively	−0.32; 0.24
Hunter (2015)	Change in prevalence of AST (measured with swipe card/self-report)	−0.61; 0.34
Multi-component interventions		
Christiansen (2014)	Change in odds of engaging in AST	0.13
Xu (2015)	Change in odds of engaging in AST	0.45
Curriculum-based interventions		
McMinn (2012)	Difference in commuting steps and MVPA between intervention and control groups	0.06/-0.03
McMinn (2012)	Difference in daily steps and MVPA between intervention and control groups	0.52/0.46
Villa-Gonzalez (2016)	Changes in weekly number of active trips	0.40
Drop-off spots		
Vanwolleghem (2014)	Change in frequency of AST	0.75
Crossing guards		
Gutierrez (2014)	Change in number of students engaging in AST	0.03

*AST* active school transportation, *MVPA* moderate-to-vigorous physical activity. Effect sizes were computed as detailed in Additional file 2. Some studies appear more than once because they have multiple measures of effect size. Cohen’s d could not be computed for 5 interventions because insufficient information was provided by the authors. Following Cohen’s^28^ guidelines, effect size can be categorized as trivial (*d* < 0.2), small (*d* = 0.2), medium (*d* = 0.5), or large (*d* = 0.8)

### Moderators and mediators

Thirteen studies examined potential moderators. Hinckson et al. [17, 18] noted that longer follow-up periods, smaller school size, higher school SES, and higher pre-intervention rate of AST predicted higher rates of AST at follow-up. Safe Routes to School interventions using multiple strategies (as defined by the 6P model) achieved larger increases in AST [42, 43], and a longer follow-up period was also associated with more substantial increases in AST [43]. In contrast, a short follow-up period was discussed as a potential reason for the lack of a significant mode shift in other interventions [20, 46]. Mammen and colleagues [19] reported that parents of older students, those living closer to school and attending urban or suburban schools (relative to rural) were more likely to report “driving less” following the implementation of an STP. Of the potential moderators examined by Stewart et al. [48], only the percentage of students cycling at baseline was negatively associated with changes in cycling. In addition, Mendoza and colleagues’ [45] results suggest that greater acculturation, more positive parental self-efficacy and outcome expectations may facilitate children’s engagement in AST.

Goodman and colleagues [36] intended to assess children’s participation in cycle training as a mediator of the relationship between exposure to the Bikeability program at the school level and children’s cycling behavior. However they found a similar frequency of cycling among children exposed and unexposed to the program. No other study described formal mediation analyses.

## Discussion

We have provided a comprehensive update on the effectiveness of AST interventions among children and adolescents. Our search strategy identified 27 papers, describing the findings of 30 distinct interventions, which have been published since the previous review [24]. Included interventions were quite diverse and changes in travel behaviors varied markedly across interventions. Included studies suggest that interventions with longer follow-up periods may achieve greater modal shifts. These observations are of particular importance for policy-makers and practitioners implementing AST interventions.

Two large SRTS interventions found that interventions including both educational activities and infrastructure changes resulted in greater increases in AST than interventions using only one of these strategies [42, 43]. These results are consistent with social-ecological models that posit that behavior is determined by multiple levels of influence including individual, interpersonal, community, policy and built environment factors [52, 53].

We noted that few interventions targeted secondary school students. As the correlates of AST may differ by age [54], one should not assume that interventions that are effective among children will work as well with adolescents. Adolescents generally have higher independent mobility [55] and, as such, the influence of parental perceptions on their school travel mode may be weaker. However, adolescents may have less favorable attitudes toward AST [56, 57], and this might be a key factor to address for interventions in secondary schools.

In the previous systematic review [24], all studies were rated as “weak” based on the EPHPP tool. In our review, 10% of the studies were rated “moderate” (even with a stricter interpretation of the blinding component of EPHPP) and, when the blinding component was dismissed as unfeasible, 30% of the studies were rated as “moderate” or “strong”. This suggests a marginal improvement in study quality over the last 6 years; however the overall quality of evidence as assessed with the GRADE approach remains low. Our sensitivity analysis shows that the blinding component exerted a floor effect on quality scores. Because all interventions received a “weak” rating for blinding, they could not be rated higher than “moderate”. Future improvement in quality ratings could be made by controlling for confounders and by using valid and reliable measures of AST, which have been reviewed elsewhere [58].

The calculated effect sizes for most interventions were trivial to small based on Cohen’s [28] thresholds. Although these widely-used thresholds are arbitrary, we have used them in the absence of alternative options. Given the large reach of interventions such as SRTS and STP, an effect size labeled as “trivial-to-small” may still be highly relevant from a population health perspective. Interestingly, a pooled intervention effect of *d* = 0.12 was obtained in a meta-analysis of 30 controlled trials on PA interventions among children and adolescents [59].

Furthermore, while our review focused specifically on the effect of interventions on travel behaviors, some included interventions have documented positive changes in other important outcomes such as children’s cycling skills [35], safe street crossing behaviors [37], attitudes toward AST [40], and higher daily PA [44, 45]. Substantial reductions in road traffic injuries among children have also been noted following implementation of SRTS [15]. More broadly, it has been proposed that interventions such as SRTS may benefit the larger communities in which they are implemented, and not only children [60].

### Mediators and moderators

A better understanding of the mediators and moderators of AST interventions could help identify what works for whom and why [61, 62]. Of particular interest, many studies emphasized the importance of having long term follow-ups given that implementation of complex AST interventions may require a substantial amount of time [17–20, 43, 46]. Similarly, qualitative evaluations focusing on the implementation of AST interventions also identify lack of time as a key challenge [63, 64]. To address the issue of follow-up length, some authors suggested that granting agencies should be encouraged to provide more long term funding [63, 64].

While there has been increased interest in studying moderators of AST interventions, none of the included studies conducted formal mediation analyses and most interventions did not include an explicit theoretical framework. Given the important role of parents in travel mode decision making [65], interventions that increase road safety may be more effective if they also target parents’ self-efficacy in allowing their child to engage in AST [45].

### Implementation of interventions

Understanding the implementation of complex AST interventions may provide valuable information for the reader to contextualize the effectiveness of such interventions. This may be particularly important for interventions such as SRTS and STP that are essentially evaluated as “natural experiments” [66] because in most cases, exposure to the intervention is not under the control of the investigators. This is a threat to internal validity because the fidelity of implementation varies, but at the same time, it represents more closely how an intervention is implemented in the “real world”. Many interventions included in this review reported that implementation varied substantially between schools [19, 20, 32, 34, 46], and in some cases, planned changes were not implemented as scheduled [32, 37, 46]. Crawford and Garrard [34] also reported that the implementation of the Ride2School program was affected by the motivation of school communities. Such challenges and discrepancies may bias our results toward the null hypothesis.

Lack of resources or unequal access to resources has been noted by many authors as a limitation to AST interventions [32, 63, 64]. In Canada, STPs and WSBs are implemented by non-governmental organizations and lack of support from provincial and federal governments has been identified as a major barrier [64]. In Texas, stakeholders expressed difficulty in navigating the SRTS regulatory process and emphasized that access to SRTS funding was very challenging for low income communities given that no up-front funding was provided [63]. More generally, WSBs typically rely on volunteers which often makes long term sustainability challenging [23, 67]. Providing paid WSB leaders may help overcome this issue.

### Strengths and limitations

As in the previous review [24], we noted that many included studies did not include a control group. Another limitation is that the original EPHPP tool seems better suited to assess studies where the unit of allocation is the individual. To address this issue, we have modified the tool so that the questions are more relevant to school-based interventions (see Additional file 1). Nevertheless, like other quality assessment tools, the scoring system of the EPHPP is rigid and may not always distinguish more robust studies from weaker ones [68]. For example, in our review, no study reported that outcome assessors were blinded, creating a floor effect whereby no intervention can be rated higher than “moderate”. Notwithstanding the importance of blinding in preventing observer bias and Hawthorne effects, a quality assessment tool should be able to discriminate stronger studies from weaker ones. Our sensitivity analysis without the blinding component of the EPHPP intended to address this issue. We acknowledge that the use of a different quality assessment tool could have resulted in different ratings of study quality as observed previously [68]. Finally, the large heterogeneity in the measurement and operationalization of AST precluded meta-analysis. The development of a standard measurement protocol may help address this issue.

The rigorous systematic review process is an important strength of the study. We followed the same search strategy as Chillón and colleagues [24] and computed standardized effect sizes which should help readers interpret the effectiveness of interventions and perform sample size calculations. Finally, the discussion of moderators, mediators and factors related to implementation should help researchers refine current interventions.

## Conclusions

The present systematic review highlights the diversity of interventions that have been implemented to promote AST in the last few years, and shows that travel behavior change varied markedly between interventions. Many interventions have shown significant increases in AST, but caution is required in interpretation given the low quality of evidence. This underscores a need for interventions using stronger study designs.

Our findings have implications for researchers and practitioners. First, it may take time for interventions to have an effect on children’s travel behaviors. Therefore, follow-ups of at least 2 years should be conducted when possible to minimize the risk of type II error. Second, while many authors indicated that implementation of interventions varied markedly across schools, it is unclear how this variation may influence effectiveness. Hence future research should examine the potential moderating effect of implementation. The fact that some interventions were not implemented as planned suggests that some of the effect sizes reported herein may be conservative. Third, there remains a clear need for investigation of the mediators of travel behavior change.

Only three interventions included some high schools, highlighting a need for more research intervening in secondary school settings. This is important given that the factors associated with AST may differ markedly between children and adolescents. Finally, because some children may live too far from their school, interventions aiming to promote active transportation to/from other destinations such as parks, shops, sport venues, and friends’ and relatives’ houses may also be warranted [69].

## Additional files

Additional file 1: Appendix 1. Adjusted criteria for the Effective Public Health Practice Project quality assessment tool for quantitative studies. (DOCX 33 kb)

Additional file 2: Appendix 2. Computation of effect sizes. (DOCX 34 kb)

## Abbreviations

AST: Active school transport
EPHPP: Effective public health practice project
GRADE: Grades of recommendation, assessment, development, and evaluation
PA: Physical activity
SRTS: Safe routes to school
STP: School travel plans
UK: United Kingdom
US: United States
WSB: Walking school buses
