# Supplementary material for: Effectiveness of active school transport interventions: a systematic review and update
Source: BMC Public Health. 2018 Feb 1;18:206. doi: 10.1186/s12889-017-5005-1 (PMC5796594; doi:10.1186/s12889-017-5005-1)
Supplement: Supplementary file 1 — Appendix 1. Adjusted criteria for the Effective Public Health Practice Project quality assessment tool for quantitative studies. (DOCX 33 kb) [file 12889_2017_5005_MOESM1_ESM.docx]

**Appendix 1. Adjusted criteria for the Effective Public Health Practice Project quality assessment tool for quantitative studies**

**A) SELECTION BIAS**

***Adaptation: for studies in which the unit of analysis is the individual, this component should be rated as specified in the original instrument. However, if the unit of analysis is the school, this component should be rated as per Chillón et al. (2011).***

**(Q1) Are the individuals selected to participate in the study likely to be representative of the target population?**

1 Very likely

2 Somewhat likely

3 Not likely

4 Can’t tell

Note: Participants are more likely to be representative of the target population if they are randomly selected from a comprehensive list of individuals in the target population (score very likely). They may not be representative if they are referred from a source (e.g. clinic) in a systematic manner (score somewhat likely) or self-referred (score not likely).

**(Q2) What percentage of selected individuals agreed to participate?**

1 80 - 100% agreement

2 60 - 79% agreement

3 Less than 60% agreement

4 Not applicable

5 Can’t tell

Note: Refers to the % of subjects in the control and intervention groups that agreed to participate in the study before they were assigned to intervention or control groups. *(this should be interpreted as the % of subjects or organizational units (e.g., schools) who provided consent, and not as the overall response rate.) If the number of individuals or units (schools, etc.) who were invited to participate is not stated, the item should be scored “Can’t tell”*

**Section rating:**

Strong (1): The selected individuals are very likely to be representative of the target population (Q1 is 1) and there is greater than 80% participation (Q2 is 1).

Moderate (2): The selected individuals are at least somewhat likely to be representative of the target population (Q1 is 1 or 2); and there is 60 - 79% participation (Q2 is 2). ‘Moderate’ may also be assigned if Q1 is 1 or 2 and Q2 is 5 (can’t tell).

Weak (3): The selected individuals are not likely to be representative of the target population (Q1 is 3); **or** there is less than 60% participation (Q2 is 3) **or** selection is not described (Q1 is 4); and the level of participation is not described (Q2 is 5).

**B) STUDY DESIGN**

***This component should be rated as specified in the original instrument. For the purpose of this quality assessment, randomized controlled trials and controlled trials in which the school (or classroom) is the unit of allocation should be rated as “strong”.***

In this section, raters assess the likelihood of bias due to the allocation process in an experimental study. For observational studies, raters assess the extent that assessments of exposure and outcome are likely to be independent. Generally, the type of design is a good indicator of the extent of bias. In stronger designs, an equivalent control group is present and the allocation process is such that the investigators are unable to predict the sequence.

**Indicate the study design:**

1 **Randomized controlled trial**: An experimental design where investigators randomly allocate eligible people to an intervention or control group. A rater should describe a study as an RCT if the randomization sequence allows each study participant to have the same chance of receiving each intervention and the investigators could not predict which intervention was next. If the investigators do not describe the allocation process and only use the words ‘random’ or ‘randomly’, the study is described as a controlled clinical trial.

Was the study described as randomized? Score YES, if the authors used words such as random allocation, randomly assigned, and random assignment. Score NO, if no mention of randomization is made. If NO, *write not applicable (N/A) for the 2 questions below and then* go to Component C.

□ No □ Yes

If Yes, was the method of randomization described? Score YES, if the authors describe any method used to generate a random allocation sequence. Score NO, if the authors do not describe the allocation method or describe methods of allocation such as alternation, case record numbers, dates of birth, day of the week, and any allocation procedure that is entirely transparent before assignment, such as an open list of random numbers of assignments. If NO is scored, then the study is a controlled clinical trial.

□ No □ Yes

If Yes, was the method appropriate? Score YES, if the randomization sequence allowed each study participant to have the same chance of receiving each intervention and the investigators could not predict which intervention was next. Examples of appropriate approaches include assignment of subjects by a central office unaware of subject characteristics, or sequentially numbered, sealed, opaque envelopes. Score NO, if the randomization sequence is open to the individuals responsible for recruiting and allocating participants or providing the intervention, since those individuals can influence the allocation process, either knowingly or unknowingly. If NO is scored, then the study is a controlled clinical trial.

□ No □ Yes

**2 Controlled clinical trial:** An experimental study design where the method of allocating study subjects to intervention or control groups is open to individuals responsible for recruiting subjects or providing the intervention. The method of allocation is transparent before assignment, e.g. an open list of random numbers or allocation by date of birth, etc.

**3 Cohort analytic (two group pre + post)**: An observational study design where groups are assembled according to whether or not exposure to the intervention has occurred. Exposure to the intervention is not under the control of the investigators. Study groups might be non-equivalent or not comparable on some feature that affects outcome.

**4 Case-control**: A retrospective study design where the investigators gather ‘cases’ of people who already have the outcome of interest and ‘controls’ who do not. Both groups are then questioned or their records examined about whether they received the intervention exposure of interest.

**5 Cohort (one group pre + post (before and after))**: The same group is pretested, given an intervention, and tested immediately after the intervention. The intervention group, by means of the pretest, act as their own control group.

**6 Interrupted time series**: A time series consists of multiple observations over time. Observations can be on the same units (e.g. individuals over time) or on different but similar units (e.g. student achievement scores for particular grade and school). Interrupted time series analysis requires knowing the specific point in the series when an intervention occurred.

**7 Other specify ____________________________**

**8 Can’t tell**

**Section rating:**

Strong (1): will be assigned to those articles that described RCTs and CCTs.

Moderate (2): will be assigned to those that described a cohort analytic study, a case control study, a cohort design, or an interrupted time series.

Weak (3): will be assigned to those that used any other method or did not state the method used.

**C) CONFOUNDERS**

By definition, a confounder is a variable that is associated with the intervention or exposure and causally related to the outcome of interest. Even in a robust study design, groups may not be balanced with respect to important variables prior to the intervention. The authors should indicate if confounders were controlled in the design (by stratification or matching) or in the analysis. If the allocation to intervention and control groups is randomized, the authors must report that the groups were balanced at baseline with respect to confounders (either in the text or a table). *Following Chillón et al. (2011), if the study did not have a control group, the assessment was not applicable.*

**(Q1) Were there important differences between groups prior to the intervention?**

1 Yes

2 No

3 Can’t tell

*4 N/A*

The following confounders were deemed to be of particular importance in the context of active transportation interventions: age, gender/sex, SES (education, income, car ownership), school, and travel behaviour pre-intervention.

**(Q2) If yes, indicate the percentage of relevant confounders that were controlled (either in the design (e.g. stratification, matching) or analysis)?**

1 80 – 100% (most)

2 60 – 79% (some)

3 Less than 60% (few or none)

4 Can’t Tell

*5 N/A*

**Section rating:**

Strong (1): will be assigned to those articles that controlled for at least 80% of relevant confounders (Q1 is 2); **or** (Q2 is 1).

Moderate (2): will be given to those studies that controlled for 60 – 79% of relevant confounders (Q1 is 1) **and** (Q2 is 2).

Weak (3): will be given when less than 60% of relevant confounders were controlled (Q1 is 1) **and** (Q2 is 3) **or** control of confounders was not described (Q1 is 3) **and** (Q2 is 4).

*Not applicable: will be given if Q1 is 4 and Q2 is 5. N/A will be given a weak (3) rating.*

**D) BLINDING^[[1]](#footnote-1)^**

**(Q1) Was (were) the outcome assessor(s) aware of the intervention or exposure status of participants?** Assessors should be described as blinded to which participants were in the control and intervention groups. The purpose of blinding the outcome assessors (who might also be the care providers) is to protect against detection bias.

1 Yes

2 No

3 Can’t tell

**(Q2) Were the study participants aware of the research question?** Study participants should not be aware of (i.e. blinded to) the research question. The purpose of blinding the participants is to protect against reporting bias.

1 Yes

2 No

3 Can’t tell

*4 Not applicable (unfeasible)*

**Section rating:**

Strong (1): The outcome assessor is not aware of the intervention status of participants (Q1 is 2) and the study participants are not aware of the research question (Q2 is 2).

Moderate (2): The outcome assessor is not aware of the intervention status of participants (Q1 is 2); or the study participants are not aware of the research question (Q2 is 2);

Weak (3): he outcome assessor is aware of the intervention status of participants (Q1 is 1); and the study participants are aware of the research question (Q2 is 1); **~~or~~** ~~blinding is not described (Q1 is 3 and Q2 is 3).^[[2]](#footnote-2)^~~

**E) DATA COLLECTION METHODS**

Tools for primary outcome measures must be described as reliable and valid. If content’ validity has been demonstrated, this is acceptable.^[[3]](#footnote-3)^ Some sources from which data may be collected are described below: Self-reported data includes data that is collected from participants in the study (e.g. completing a questionnaire, survey, answering questions during an interview, etc.). Assessment/Screening includes objective data that is retrieved by the researchers. (e.g. observations by investigators). Medical Records/Vital Statistics refers to the types of formal records used for the extraction of the data. **Reliability and validity can be reported in the study or in a separate study. For example, some standard assessment tools have known reliability and validity.**

**(Q1) Were data collection tools shown to be valid?**

1 Yes

2 No, or Can’t tell.

**(Q2) Were data collection tools shown to be reliable?**

1 Yes

2 No, or Can’t tell.

*The primary outcome measure for the present review is children’s travel behaviour.*

**Section rating:**

Strong (1): The data collection tools have been shown to be valid (Q1 is 1); **and** the data collection tools have been shown to be reliable (Q2 is 1).

Moderate (2): The data collection tools have been shown to be valid (Q1 is 1); **and** the data collection tools have not been shown to be reliable or reliability is not described (Q2 is 2)

Weak (3): The data collection tools have not been shown to be valid, or validity was not reported (Q1 is 2) or both reliability and validity are not described~~.~~

**F) WITHDRAWALS AND DROP-OUTS**

*For studies in which the* ***unit of analysis*** *is the individual, this component should be rated as specified in the original instrument. However, if the unit of analysis is the school, this component should be rated at the school level. Following Chillón et al. (2011), if the study had only 1 measure (pre or post), then the assessment was not applicable (N/A). Withdrawal/drop-out is defined as individuals (or units/schools, etc.) who did not complete the study after agreeing to participate. This is distinct from non-recruitment (i.e, not agreeing to participate). Individuals or units who did not complete assessments are defined as having dropped out or withdrawn, regardless of whether they participated in the program.*

**(Q1) Were withdrawals and drop-outs reported in terms of numbers and/or reasons per group?**

1 Yes

2 No

3 Can’t tell

4 Not Applicable (i.e. one time surveys or interviews)

**(Q2) Indicate the percentage of participants completing the study. (If the percentage differs by groups, record the lowest).** The percentage of participants completing the study refers to the % of subjects remaining in the study at the final data collection period in all groups (i.e. control and intervention groups). *Note: the denominator is the number of participants who agreed to participate in the study.*

1 80 -100%

2 60 - 79%

3 less than 60%

4 Can’t tell

5 Not Applicable (e.g. Retrospective case-control)

**Section rating:**

Strong (1): will be assigned when the follow-up rate is 80% or greater (Q2 is 1).

Moderate (2): will be assigned when the follow-up rate is 60 – 79% (Q2 is 2) OR Q2 is 5 (N/A).

Weak (3): will be assigned when a follow-up rate is less than 60% (Q2 is 3) or if the withdrawals and drop-outs were not described (Q2 is 4).

*If Q1 is not applicable and, due to the nature of the intervention, participants can’t drop out, the rating of strong (1) will be given.*

**G) INTERVENTION INTEGRITY**

The number of participants receiving the intended intervention should be noted (consider both frequency and intensity). For example, the authors may have reported that at least 80 percent of the participants received the complete intervention. The authors should describe a method of measuring if the intervention was provided to all participants the same way. As well, the authors should indicate if subjects received an unintended intervention that may have influenced the outcomes. For example, co-intervention occurs when the study group receives an additional intervention (other than that intended). In this case, it is possible that the effect of the intervention may be over-estimated. Contamination refers to situations where the control group accidentally receives the study intervention. This could result in an under-estimation of the impact of the intervention.

**(Q1) What percentage of participants received the allocated intervention or exposure of interest?**

1 80 -100%

2 60 - 79%

3 less than 60%

4 Can’t tell

**(Q2) Was the consistency of the intervention measured?**

1 Yes

2 No, or Can’t tell

**(Q3) Is it likely that subjects received an unintended intervention (contamination or co-intervention) that may influence the results?**

1 Yes

2 No, or Can’t tell

*Following Chillón et al. (2011), if* *there was no indication about consistency in the study, it was assumed to not be reported or available and the answer to Q2 was 2 (no). If there was no indication about whether the participants received an unintended intervention that may influence the results, then it was assumed to not be available and the answer to Q3 was 2 (no).*

**H) ANALYSES**

**(Q1) Indicate the unit of allocation (circle one)**

community organization/institution practice/office individual

**(Q2) Indicate the unit of analysis (circle one)** *Following Chillón et al. (2011), the unit of allocation and analysis was organization/institution when results were organized for each school, and it was individual when results were organized without separating schools. The unit of allocation and analysis is assumed to be ordinal. In a school setting, community is interpreted as equivalent to local education authority, organization is interpreted as equivalent to school, and practice/office is interpreted as equivalent to class.*

community organization/institution practice/office individual *other*

**(Q3) Are the statistical methods appropriate for the study design? Was the quantitative analysis appropriate to the *primary^[[4]](#footnote-4)^* research question being asked?**

1 Yes

2 No

3 Can’t tell

**(Q4) Is the analysis performed by intervention allocation status (i.e. intention to treat) rather than the actual intervention received?** An intention-to-treat analysis is one in which all the participants in a trial are analyzed according to the intervention to which they were allocated, whether they received it or not. Intention-to-treat analyses are favoured in assessments of effectiveness as they mirror the noncompliance and treatment changes that are likely to occur when the intervention is used in practice, and because of the risk of attrition bias when participants are excluded from the analysis. *In many of the post hoc nonexperimental studies, intention-to-treat is also interpreted as means that all participants were analyzed regardless of the fidelity of the intervention delivery, not solely to mean inclusion of drop-outs in the analysis.*

1 Yes

2 No

3 Can’t tell

4 N/A

**I) FINAL SCORING**

**GLOBAL RATING FOR THIS PAPER (circle one):**

1 STRONG (no WEAK ratings)

2 MODERATE (one WEAK rating)

3 WEAK (two or more WEAK ratings)

**Is there a discrepancy between the two reviewers with respect to the component (A-F) ratings?**

□ No

□ Yes

**If yes, indicate the reason for the discrepancy:**

1 Oversight

2 Differences in interpretation of criteria

3 Differences in interpretation of study

**Final decision of both reviewers (circle one):**

1 STRONG 2 MODERATE 3 WEAK

**References**

1. Chillón P, Evenson KR, Vaughn A, Ward DS. A systematic review of interventions for promoting active transportation to school. Int J Behav Nutr Phys Act. 2011;8:10. <http://www.biomedcentral.com/content/pdf/1479-5868-8-10.pdf>
2. Effective Public Health Practice Project: Quality Assessment Tool for Quantitative Studies. Available at: <http://www.ephpp.ca/PDF/Quality%20Assessment%20Tool_2010_2.pdf>

1. In the majority of studies assessed, blinding of participants was not feasible given the design of the intervention. Most studies did not provide any detail about blinding, and in some cases (i.e. walking school buses), blinding of assessors was impractical. Because the blinding component created a ceiling effect where no study could be rated higher than moderate, the review team decided to also report a sensitivity analysis that excluded the blinding component. [↑](#footnote-ref-1)
2. We believe that a “moderate” rating should not be given when blinding is not reported because this would be equivalent to giving “bonus points” for poor quality of reporting. [↑](#footnote-ref-2)
3. Face validity provides very weak evidence of validity, and therefore it was considered unacceptable. [↑](#footnote-ref-3)
4. If an appropriate method is used for the primary research question, but an inappropriate method is used for a secondary question, this item was still rated as a “yes”. [↑](#footnote-ref-4)
