# Supplementary material for: Effectiveness of active school transport interventions: a systematic review and update
Source: BMC Public Health. 2018 Feb 1;18:206. doi: 10.1186/s12889-017-5005-1 (PMC5796594; doi:10.1186/s12889-017-5005-1)
Supplement: Supplementary file 2 — Appendix 2. Computation of effect sizes. (DOCX 34 kb) [file 12889_2017_5005_MOESM2_ESM.docx]

**Appendix 2. Computation of effect sizes**

| **Lead author (year)** | **Calculation of Cohen’s d** | | | |
| --- | --- | --- | --- | --- |
|  | **Conceptual data** | **Numerical data** | **Formula** | **Cohen’s d** |
| Buckley (2013) – fall event | Change in the number of students and parents observed engaging in AST between baseline and follow-up | The authors report a 101% increase in AST but numbers are not provided and it is unclear how many students attended the schools, so effect size cannot be computed | N/A | N/A |
| Buckley (2013) – spring event | Change in the number of students and parents observed engaging in AST between baseline and follow-up | The authors report significant changes in AST (χ^2^ = 11.6; p = 0.009), but is unclear how many students attended the schools, so effect size cannot be computed | N/A | N/A |
| Buliung (2011) | Change in the proportion of children who reported engaging in AST between baseline and follow-up | The mode share of AST increased from 43.8 to 45.9% and 5012 children attended the 12 participating schools | OR (for proportions)† = (p_1_ * (1 - p_2_)) / (p_2_ * (1 - p_1_))  d = ln OR * √3/π  (Borenstein et al., 2009; Lipsey & Wilson, 2001) | d = 0.05 (trivial) |
| Bungum (2014) | Change in the number of students observed engaging in AST between baseline and follow-up | χ^2^ = 27.2; N=1,336 students | d = 2 * √(χ^2^/(N- χ^2^))  (Lipsey & Wilson, 2001) | d = 0.29 (small) |
| Christiansen (2014) | Change in the mode share of AST in the exp. vs. con. group | OR = 1.27 | d = ln OR * √3/π  (Borenstein et al., 2009) | d = 0.13 (trivial) |
| Coombes (2016) | Change in the frequency of AST in the exp. vs. con. group between baseline and follow-up | Proportions of school trips using AST at baseline, mid-intervention and post-intervention in the con. group: 0.638; 0.783; 0.621 vs. in the exp. group: 0.501; 0.533; 0.591. | OR (for proportions) = (p_1_ * (1 - p_2_)) / (p_2_ * (1 - p_1_))  d = ln OR * √3/π  (Borenstein et al., 2009; Lipsey & Wilson, 2001) | At mid-intervention: d = -0.32 (small)  At post-intervention: d = 0.24 (small) |
| Crawford (2013) – pilot schools | Change in: 1) the number of students observed engaging in AST; and 2) the proportion of children who reported engaging in AST at baseline and follow-up in the exp. vs. con. group. Analyses stratified by type of urbanization (inner suburban and outer suburban) | Observation counts [inner suburban]: exp. (pre: 31.4% AST; post: 39.0% AST) and con. (pre: 40.8% AST; post: 36.8% AST). [outer suburban]: exp. (pre: 18.9% AST; post: 19.7% AST) and con. (pre: 24.6% AST; post: 29.8% AST).  Child report [inner suburban]: exp. (pre: 43.2% AST; post: 52.0% AST) and con. (pre: 50.1% AST; post: 45.8% AST). [outer suburban]: exp. (pre: 26.6% AST; post: 25.0% AST) and con. (pre: 34.6% AST; post: 31.2% AST). | OR (for proportions) = (p_1_ * (1 - p_2_)) / (p_2_ * (1 - p_1_))  d = ln OR * √3/π  (Borenstein et al., 2009; Lipsey & Wilson, 2001) | Observation counts: inner suburban: d = 0.27 (small). Outer suburban: d = -0.12 (trivial). Hands-up survey: inner suburban: d = 0.30 (small); outer suburban: exp: d = 0.04 (trivial) |
| Crawford (2013) – program schools | Change in the mode share of AST assessed either with child- or parent-report from baseline to follow-up | Parent-report: baseline (n=410; 47.9% AST) vs. follow-up (n=358; 49.6% AST)  Child-report: baseline (n=479; 51.1% AST) vs. follow-up (n=403; 48.7% AST) | OR (for proportions) = (p_1_ * (1 - p_2_)) / (p_2_ * (1 - p_1_))  d = ln OR * √3/π  (Borenstein et al., 2009; Lipsey & Wilson, 2001) | Parent surveys: d = 0.04 (trivial); child surveys: d = -0.06 (trivial) |
| Ducheyne (2014) | Change in weekly time spent cycling to/from school (in minutes) from pre-test to post-test in 3 groups: con, intervention and intervention + parent | Intervention group (n=25): pre-test (19.6 ± 27.1), post-test (26.7 ± 28.9)  Intervention + parent group (n=34): pre-test (24.4 ± 30.6), post-test (20.0 ± 32.0)  Control group (n=35): pre-test (15.5 ± 29.7), post-test (10.2 ± 23.0) | d = (X_1_ – X_2_)/sd_pooled_  Cohen (1988) – *d* is reported for the interaction between time and group | Intervention vs. control group: d = 0.46 (small); intervention + parent vs. control: d = 0.03 (trivial) |
| Goodman (2016) | Difference in the likelihood of cycling to school between children exposed and unexposed to bikeability at the school and individual level | School level: OR = 0.73  Individual level: OR = 1.38 | d = ln OR * √3/π  (Borenstein et al., 2009; Lipsey & Wilson, 2001) | School level: d = -0.17 (trivial)  Individual level: d = 0.18 (trivial) |
| Gutierrez (2014) | Change in the number of children observed engaging in AST in the exp. vs. con. group | Exp. group (n=34 intersections): pre-test (41.67 ± 29.83), post-test (43.15 ± 33.22)  Con. group (n=24 intersections): pre-test (34.81 ± 33.41), post-test (35.19 ± 36.34) | d = (X_1_ – X_2_)/sd_pooled_  *d* is reported for the interaction between time and group | d = 0.03 (trivial) |
| Henderson (2013) | Change in the mode share of AST from baseline to follow-up for the morning and afternoon trips | The mode share of AST increased from 18% to 42% for the morning trip and from 18% to 23% for the afternoon trip | OR (for proportions) = (p_1_ * (1 - p_2_)) / (p_2_ * (1 - p_1_))  d = ln OR * √3/π  (Borenstein et al., 2009; Lipsey & Wilson, 2001) | Morning trip: d = 0.66 (medium)  Afternoon trip: d = 0.17 (small) |
| Hinckson (2011a) | Change in the mode share of AST from baseline to follow-up | Proportion of active trips at baseline (34.9%) and follow-up (40.8%). | OR (for proportions) = (p_1_ * (1 - p_2_)) / (p_2_ * (1 - p_1_))  d = ln OR * √3/π  (Borenstein et al., 2009; Lipsey & Wilson, 2001) | d = 0.14 (trivial) |
| Hinckson (2011b) | Change in the mode share of AST from baseline to follow-up after 1, 2 and 3 years following implementation of school travel plan | 1 year: OR = 0.73; 2 years: OR = 2.53; 3 years: OR = 2.65 | d = ln OR * √3/π  (Borenstein et al., 2009) | Effect after 1 year (d = -0.17; trivial), 2 years (d = 0.51; moderate), and 3 years (d = 0.54; moderate) |
| Hoelscher (2016) | Change in the proportion of children engaging in AST between intervention and control groups | Data was only provided in the form of a Figure, so effect size cannot be computed | N/A | N/A |
| Hunter (2015) | Change in the mode share of active transportation from baseline to follow-up measured with a swipe card technology and a child survey | Swipe card technology (n=3817): baseline (29%), follow-up (12%)  Self-report (n=781): baseline (77%), follow-up (86%) | OR (for proportions) = (p_1_ * (1 - p_2_)) / (p_2_ * (1 - p_1_))  d = ln OR * √3/π  (Borenstein et al., 2009; Lipsey & Wilson, 2001) | Swipe card technology: d = -0.61 (moderate); self-report: d = 0.34 (small) |
| Johnson (2015) - Bikeability | Difference in the odds of cycling to school between children who received Bikeability training and those who did not | OR = 2.25 | d = ln OR * √3/π  (Borenstein et al., 2009) | d = 0.45 (small) |
| Johnson (2015) - CensusAtSchool | Difference in the odds of cycling to school between children who received Bikeability training and those who did not | OR = 1.60 | d = ln OR * √3/π  (Borenstein et al., 2009) | d = 0.26 (small) |
| Mammen (2014a) | Change in school travel habits following the implementation of STPs | ~17% of parents reported “driving less” as a result of the intervention in the morning and afternoon. Effect size cannot be computed because baseline data are unavailable. | N/A | N/A |
| Mammen (2014b) | Changes in the mode share of AST from baseline to follow-up | Baseline rates of AST were 27.8% in the morning and 31.3% in the afternoon. Follow-up rates were 27.2% and 31.7% (G. Mammen, personal communication) | OR (for proportions) = (p_1_ * (1 - p_2_)) / (p_2_ * (1 - p_1_))  d = ln OR * √3/π  (Borenstein et al., 2009; Lipsey & Wilson, 2001) | Morning: d = -0.02 (trivial); afternoon: d = 0.01 (trivial) |
| McDonald (2013) | Changes in the likelihood of AST in schools with a Safe Routes to School program and control schools | No baseline data provided, so impossible to compute effect size | N/A | N/A |
| McDonald (2014) | Changes in the likelihood of AST in schools with a SRTS program and control schools | 18% of children engaged in AST before SRTS and the adjusted proportion was 23.5% after 5 years. In schools with only infrastructure changes, the proportion was 21.3% after 5 years. In schools with only education / encouragement interventions, it was 22.5% after 5 years. | OR (for proportions) = (p_1_ * (1 - p_2_)) / (p_2_ * (1 - p_1_))  d = ln OR * √3/π  (Borenstein et al., 2009; Lipsey & Wilson, 2001) | Overall: d = 0.19 (trivial), infrastructure only: d = 0.12 (trivial), education / enforcement only: d = 0.15 (trivial) |
| McMinn (2012) | Changes in the number of steps taken and minutes of MVPA during the commute to/from school and in the whole day in the exp. relative to the con. group | Commuting steps: exp.: pre-test (2395 ± 936) and post-test (2124 ± 852) vs. con.: pre-test (2186 ± 1091) and post-test (1861 ± 953). Daily steps: exp.: pre-test (10766 ± 3370) and post-test (9865 ± 3494) vs. con.: pre-test (12013 ± 9485) and post-test (9485 ± 2600). Commuting MVPA (sec.): pre-test (1082 ± 499) and post-test (924 ± 415) vs. con.: pre-test (1016 ± 551) and post-test (870 ± 470). Daily MVPA (sec.): pre-test (5062 ± 1644) and post-test (4633 ± 1722) vs. con.: pre-test (5827 ± 1664) and post-test (4656 ± 1431). | d = (X_1_ – X_2_)/sd_pooled_  *d* is reported for the interaction between time and group | Commuting steps: d = 0.06 (trivial); daily steps: d = 0.52 (moderate); commuting MVPA: d = -0.03 (trivial); daily MVPA: d = 0.46 (small) |
| Mendoza (2011) | Changes in the percentage of trips made by AST and PA in WSB participants relative to controls | Exp. children increased their weekly percent AST from 23.8% ± 9.2% at baseline to 54.0% ± 9.2% at follow-up, whereas con. children decreased their weekly percent AST from 40.2% ± 8.9% to 32.6% ± 8.9%. Exp. children increased their MVPA from 46.6 ± 4.5 to 48.8 ± 4.5 min/day while con. decreased theirs from 46.1 ± 4.3 to 41.3 ± 4.3 min/day. Note: data originally reported as mean ± SE, so we needed to convert the SEs to SDs prior to calculating the effect size. | For AST: OR (for proportions) = (p_1_ * (1 - p_2_)) / (p_2_ * (1 - p_1_))  d = ln OR * √3/π  (Borenstein et al., 2009; Lipsey & Wilson, 2001)  For MVPA:  SD = SE * √N  d = (X_1_ – X_2_)/sd_pooled_  *d* is reported for the interaction between time and group | % of trips made by AST: d = 0.40 (small). MVPA: d = 0.18 (trivial) |
| Østergaard (2015) | Changes in the number of weekly trips made by AST in the exp. relative to the con. group | Unstandardized regression coefficient (B) = 0.15 (95% CI = -0.25; 0.54). Sample size: N=2401, with exp. (n=1296) and con. (n=1105) groups | SD = √N * (upper limit of 95% CI - lower limit of 95% CI / 3.92)  X_1_ – X_2_ ≈ B  (Lipsey & Wilson, 2001)‡ | d = 0.02 (trivial) |
| Sayers (2012) | Difference in the percentage of time spent in MVPA across the week between WSB participants and controls | WSB group (n=38): 20.9 ± 6.9%; Controls (n=39): 23.4 ± 8.8% | d = (X_1_ – X_2_)/sd_pooled_  *d* is reported for the difference between groups | d = -0.32 (small) |
| Stewart (2014) | Change in the mode share of AST before and after implementation of SRTS | Prevalence of AST at baseline: 12.8 ± 11.2; at follow-up: 19.8 ± 16.4. Prevalence of walking: at baseline: 8.8 ± 8.2; at follow-up: 13.3 ± 11.2. Prevalence of cycling at baseline: 2.0 ± 3.2; at follow-up: 3.2 ± 4.2. | OR (for proportions) = (p_1_ * (1 - p_2_)) / (p_2_ * (1 - p_1_))  d = ln OR * √3/π  (Borenstein et al., 2009; Lipsey & Wilson, 2001) | AST: d = 0.28 (small); walking: d = 0.26 (small); cycling: d = 0.27 (small) |
| Vanwolleghem (2014) | Difference in the number of weekly active school trips before and after implementation of drop-off spots | Mean number of active school trips at baseline: 1 ± 2 and follow-up: 3 ± 2. Assuming 10 schools journeys across the week, the proportion of active trips increased from 10 to 30%. | OR (for proportions) = (p_1_ * (1 - p_2_)) / (p_2_ * (1 - p_1_))  d = ln OR * √3/π  (Borenstein et al., 2009; Lipsey & Wilson, 2001) | d = 0.75 (medium) |
| Villa-Gonzalez (2016) | Difference in the changes in the number of active school trips between pre-test and follow-up in exp. vs. con. group | Change scores (Δ) ± SE reported for the exp. group (0.6 ± 0.2 trips) and the con. group (-0.4 ± 0.3 trips) | SD = SE * √N  X_1_ – X_2_ ≈ Δ_1_ – Δ_2_  d = (X_1_ – X_2_)/sd_pooled_  *d* is reported for the difference in change scores between groups  (Lipsey & Wilson, 2001) | d = 0.40 (small) |
| Xu (2015) | Likelihood of switching from motorized to active travel in the exp. group relative to the con. group | OR = 2.24 | d = ln OR * √3/π  (Borenstein et al., 2009) | d = 0.45 (small) |

Note: AST: active school travel; con: control; exp: experimental; MVPA: moderate-to-vigorous physical activity; STP: school travel plan; SRTS: Safe Routes to School; WSB: walking school bus. Effect size was interpreted following Cohen’s (1988) guidelines: < 0.20 = trivial; 0.20-0.49: small; 0.50-0.79: medium; ≥ 0.80: large. † In the formula for odds ratios, p_1_ represents proportions at follow-up and p_2_ represents proportions at baseline. ‡ The computation of standard deviation from a 95% confidence interval was performed as specified in the Cochrane handbook: <http://handbook.cochrane.org/chapter_7/7_7_3_2_obtaining_standard_deviations_from_standard_errors_and.htm>

**Sources and formulas**:

Borenstein M, Hedges LV, Higgins JPT, Rothstein HR. Converting among effect sizes. In: Borenstein M, Hedges LV, Higgins JPT, Rothstein HR (Eds.). *Introduction to meta-analysis*, pp. 45-49. Chichester, UK: John Wiley &

Sons; 2009. Available from: <https://www.meta-analysis.com/downloads/Meta-analysis%20Converting%20among%20effect%20sizes.pdf>

Cohen J. *Statistical power analysis for the behavioral science* (2^nd^ Ed.). Lawrence Erlbaum, Hillsdale, NJ; 1988.

Lipsey MW, Wilson DB. *Practical meta-analysis*. Thousand Oaks, CA: Sage publications; 2001. 247p.
